# Self-Healing, Flexible and Smart 3D Hydrogel Electrolytes Based on Alginate/PEDOT:PSS for Supercapacitor Applications

**DOI:** 10.3390/polym15030571

**Published:** 2023-01-22

**Authors:** Nujud M. Badawi, Mamta Bhatia, S. Ramesh, K. Ramesh, Mufsir Kuniyil, Mohammed Rafi Shaik, Mujeeb Khan, Baji Shaik, Syed F. Adil

**Affiliations:** 1 Centre for Ionics, Department of Physics, Faculty of Science, Universiti Malaya, Kuala Lumpur 50603, Malaysia; 2 Department of Physics, Acharya Narendra Dev College, University of Delhi, Delhi 110019, India; 3 Department of Chemistry, College of Science, King Saud University, P.O. Box 2455, Riyadh 11451, Saudi Arabia; 4 School of Chemical Engineering, Yeungnam University, Gyeongsan 38541, Republic of Korea

**Keywords:** flexible, self healing, smart hydrogel, electrolyte, supercapacitor

## Abstract

Hydrogel electrolytes for energy storage devices have made great progress, yet they present a major challenge in the assembly of flexible supercapacitors with high ionic conductivity and self-healing properties. Herein, a smart self-healing hydrogel electrolyte based on alginate/poly (3,4-ethylenedioxythiophene):poly(styrenesulfonate) (alginate/PEDOT:PSS)(A/P:P) was prepared, wherein H_2_SO_4_ was employed as a polymeric initiator, as well as a source of ions. PEDOT:PSS is a semi-interpenetrating network (IPN) that has been used in recent studies to exhibit quick self-healing properties with the H₂SO₃ additive, which further improves its mechanical strength and self-healing performance. A moderate amount of PEDOT:PSS in the hydrogel (5 mL) was found to significantly improve the ionic conductivity compared to the pure hydrogel of alginate. Interestingly, the alginate/PEDOT:PSS composite hydrogel exhibited an excellent ability to self-heal and repair its original composition within 10 min of cutting. Furthermore, the graphite conductive substrate-based supercapacitor with the alginate/PEDOT:PSS hydrogel electrolyte provided a high specific capacitance of 356 F g^−1^ at 100 mV/s g^−1^. The results demonstrate that the A/P:P ratio with 5 mL PEDOT:PSS had a base sheet resistance of 0.9 Ω/square. This work provides a new strategy for designing flexible self-healing hydrogels for application in smart wearable electronics.

## 1. Introduction

With the recent development of smart wearable electronics, such as portable biosensors, watches, and smart textiles, it is imperative that energy storage systems be reliable in the face of mechanical damage [1,2]. Recently, self-healing flexible supercapacitors have received increasing attention, because smart electrochemical capacitors (ECs) have high specific capacitance, energy and power densities, cycle life, and fast charge–discharge processes [3,4,5,6]. In addition, flexible hydrogel electrolytes play an essential role in the discharging and charging of supercapacitors. Self-healing polymers are often used for the synthesis of electrolytes, where good electrochemical properties are very important in supercapacitors [7]. In recent decades, supercapacitors and various types of electrolytes, including gels, aqueous, and ionic liquids with redox activity, that are suitable for large-scale manufacturing have been designed and tested. Conversely, energy storage devices that use liquid electrolytes or those based on solid polymer electrolytes require a high standard of materials for safe encapsulation, with variable geometries. This opens new opportunities for the development of energy storage devices in the field of smart wearable electronics [8]. Simultaneously, it is important for smart electrochemical capacitors to possess the self-healing properties of the hydrogel electrolyte, because it directly restores its capacitive performance when subjected to misuse, such as cutting or breakage, in record time [9,10,11].

To introduce the features of self healing and flexibility into high-performance hydrogel solutions, alginate is used as a polymeric substrate, which not only induces super-elasticity in the electrolyte, but also acts as a stable and environmentally friendly precursor [12,13,14]. There have been several reports on the use of polymeric alginate along with acidic KCl, H_3_BO_3_, KOH, and LiCl [15,16,17]. However, the use of inorganic ions to maintain the conductive nature of the polymer while inducing flexibility exerts an unfavorable effect owing to the interference of the ions within the polymeric framework. The ions adversely affect the intracellular hydrogen bonds between the conductive and flexible components of the composite polymer, resulting in poor self healing [15,18]. To deal with this problem, the polymer electrolytes must initially be solid. Furthermore, these electrolytes are to be treated with inorganic ions to be strong enough to support the massive deformation caused by the non-covalent hydrogen bonding [19] and coordination [20] bonds, thus forming flexible conductive polymer electrolytes with high curability.

The incorporation of physical (conformational alterations) and chemical (bond formation) mechanisms enhances the self-healing capabilities of maintenance-free materials [21]. Therefore, the dynamics of the interplay of movable crosslinks and reversible bonds has prompted the proposal of a composite hydrogel electrolyte, leading to its sustainable application in wearable electronics with fast self-healing properties [22].

Currently, composite polymers such as PEDOT:PSS (a good example of semi IPN) have attracted a great deal of attention, as they are used in various electrochemical devices such as fuel cells [23], dye-sensitive solar cells, and supercapacitors [24] due to their high ionic conductivity property [25,26,27]. Moreover, their flexibility and self-healing properties have been studied with the application of polyethylene glycol and alginate, and their effects on the tensile strength of the resulting conductive polymer have been reported. In addition, a study by Meen et al. [23] reported that the addition of dilute H_2_SO_4_ boosted the conductivity of the composite (PEDOT:PSS) by significantly reducing the sheet resistance. This reduction in sheet resistance was attributed to the fact that nonconductive anions of some PSSˉ were replaced by conductive anions of H_2_SO₄. They reported that this substitution reaction caused greater aggregation of PEDOT chains, favoring the enhancement of conductivity and leading to high transmittance and transparency. Their work thus indicated that H₂SO₄ doping in PEDOT:PSS films can prove useful in optoelectronic devices, in which transparent conductive electrodes find application [24]. 

Similarly, Shi et al. reported a wet process for the fabrication of electrochemical capacitors (ECs) using acid-treated (H_2_SO_4_) PEDOT:PSS as an electrode material and graphite foil as the current collector. The as-fabricated EC exhibited excellent electrochemical stability with high areal (994 μF cm^−2^) and volumetric (16.6 F cm^−3^) specific capacitances at 120 Hz [28]. The same group also reported a PEDOT:PSS-based ultrahigh-conductive polymer hydrogel that was prepared by the thermal treatment of PEDOT:PSS with H_2_SO_4_ [28]. The polymer-based hydrogel supercapacitor demonstrated high volumetric capacitance (202 F cm^−3^ at 0.54 A cm^−3^), with an extraordinarily high level of performance, which can be easily fabricated in diverse forms, including as films, fibers and columns. Additionally, PEDOT:PSS-based functional tough hydrogels have also been used in various other advanced devices for translational applications [29]. 

In this work, we report the preparation of an alginate/PEDOT:PSS hydrogel, wherein H_2_SO_4_ is employed for dual purposes—as both the polymerization initiator and the source of ions within the polymeric framework—with the aim of improving ionic conductivity, hydrogen bonding, and coordination. Thus, the use of H_2_SO_4_ led to the formation of flexible conductive polymer electrolytes with high curability. Based on the properties of conducting polymers such as PEDOT:PSS, Song et al. [30] reported their application as semitransparent supercapacitors with high conductivity via H₂SO₃ treatment. Hina et al. [31] reported the fabrication of aqueous solid-state supercapacitors based on poly(PEDOT:PSS) composite hydrogel electrolytes through free-radical polymerization. For this fabrication, they added sodium montmorillonite clay as a crosslinker. The present work demonstrates the novelty of the rapid self-healing properties of alginate/PEDOT:PSS with the addition of H₂SO₃. Notably, in comparison with pure hydrogel alginate, the addition of PEDOT:PSS enhanced the ionic conductivity to a great extent, and also showed rapid self healing within minutes of cutting.

## 2. Results and Discussion

### 2.1. Synthesis of Composite Hydrogel Electrolytes

**Figure 1:**
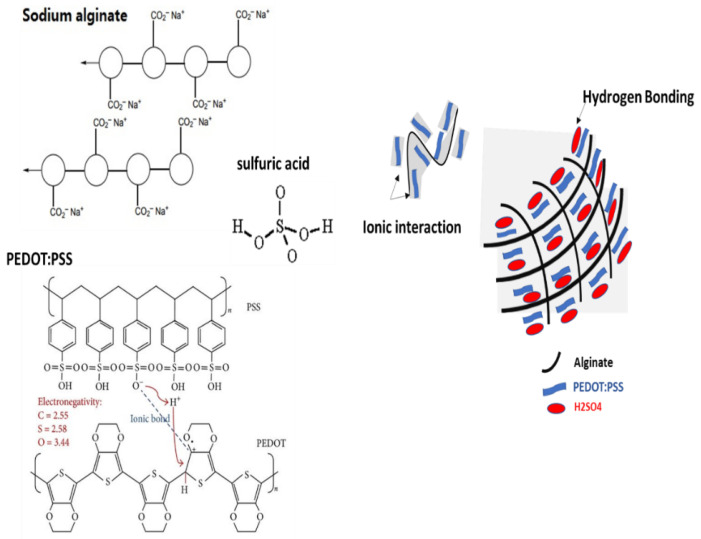
Schematic illustration of mixed ionic and electronic conduction in composite hydrogel electrolyte.

An alginate/PEDOT:PSS hydrogel electrolyte was prepared using H_2_SO_4_ as an initiator of alginate polymerization while encapsulating the PEDOT:PSS in the framework. This yielded an electrically conductive hydrogel made up of alginic acid gel with PEDOT:PSS trapped in the framework (Figure 1).

### 2.2. Physical Crosslinked Alginate/PEDOT:PSS Hydrogel

Strong interactions between chains is a distinctive feature of alginate/PEDOT:PSS hydrogels, because a stable linkage forms in the molecular network. The alginate polymer network makes the entry and retention of water in the hydrogel easier. Through non-covalent methods such as hydrogen bonding and hydrophobic forces between polymer chains, hydrogels have been created. These reactions create hydrogels, which are physically only gels with a high degree of water sensitivity. Because hydrogels do not need any harmful chaperone particles, they are safe for use in flexible wearable technology [32]. Additionally, the hydrogel contains alginate, a type of normal anionic polysaccharide. The addition of H_2_SO_4_ caused the hydrogel to crystallize, which caused the chains to be joined together by hydrogen bonding. Thus, the mechanical strength of the hydrogel increased compared to before crystallization. PEDOT:PSS affects the hydrogel characteristics. Moreover, the mechanical strength of the PEDOT:PSS hydrogel is improved by the addition of polysaccharides and alginate (Figure 1).

### 2.3. Morphology and Distribution of the Alginate/PEDOT:PSS Hydrogel Electrolyte

**Figure 2:**
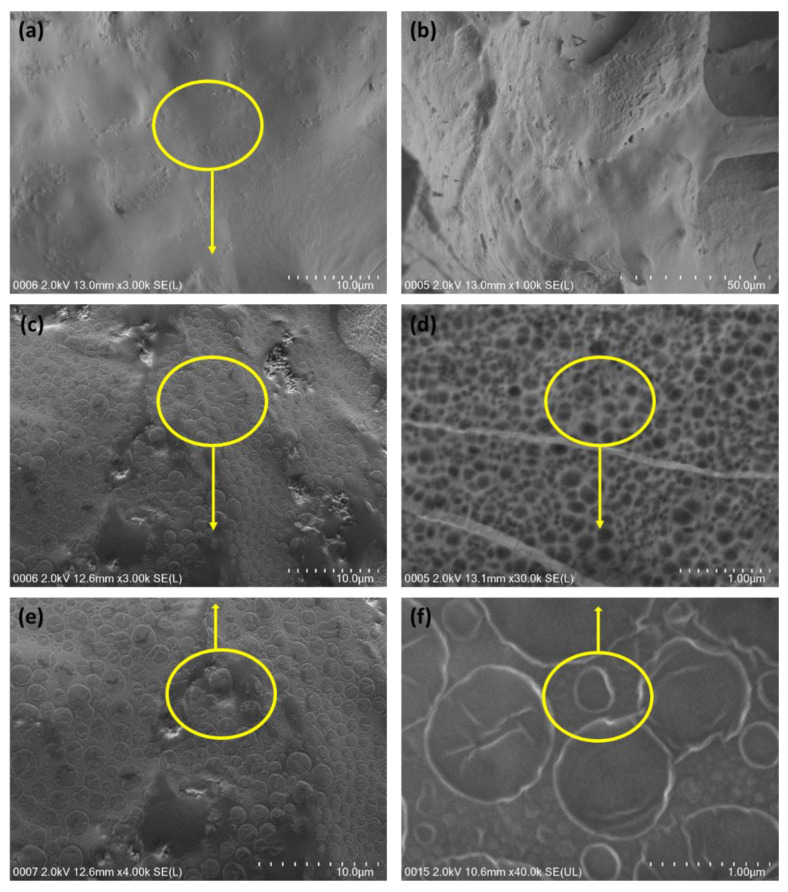
(**a**,**b**) SEM images of the alginate hydrogel and (**c**,**d**) the alginate/PEDOT:PSS hydrogel electrolyte at (**e**) low and (**f**) high magnification.

The microstructures of the alginate hydrogel and polymerized A/P:P hydrogel were studied using SEM. In the low-resolution images (Figure 2a,b), the three-dimensional morphology appears to be flaky, with visible interconnection bridges. High-resolution images (Figure 2a,b) depict spherical pores on the flake-like hydrogel surface [33]. According to the SEM images of all the samples in Figure 2, the pore diameters of the hydrogels before adding PEDOT:PSS were larger compared to A/P:P. This is because of the viscosity of PEDOT:PSS dispersion, which clogs the pores, thus reducing the pore size [33]. The displayed SEM images of hydrogels obtained by treatment with the hydrogel show a characteristic 3D fibrous network (Figure 2d–f). The alginate hydrogel surrounds PSS particles, and an organic network forms because of self-assembly in H_2_SO_4_. The nearby micelles physically intertwine via π–π stacking and H bonding of PEDOT:PSS molecules [34], as well as through polar solvents such as water.

The PEDOT:PSS molecules form micellar structures. However, the surface morphology of the alginate hydrogel changed to a foam-like microstructure (Figure 2c,d) upon polymerization catalyzed by H_2_SO_4_ in the presence of PEDOT:PSS [35]. H_2_SO_4_ catalyzes the inter-connection of the structure by forming cross-links and biphasic encapsulation of PEDOT:PSS at the hydrogel interface (Figure 2e,f) [36,37,38].

### 2.4. FT-IR Spectra 

**Figure 3:**
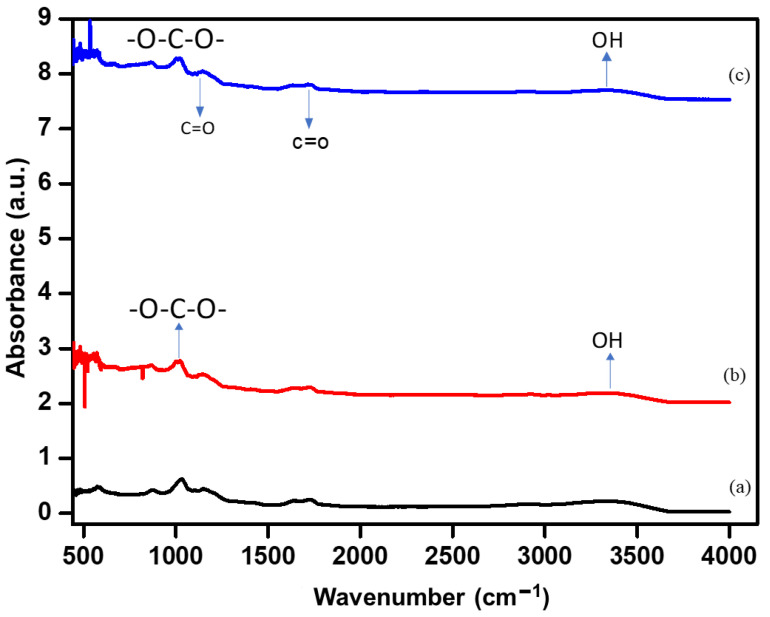
The FT-IR spectrum of the (**a**) alginate and (**b**,**c**) A/P:P hydrogel.

The chemical structure of the ‘as-synthesized’ alginate/PEDOT:PSS hydrogel electrolyte was analyzed by FT-IR spectroscopy (Figure 3). The key to flexible curable conductive hydrogels is the use of a PEDOT:PSS synthetic polymer doped in an alginate solution and its subsequent conversion into a conductive hydrogel by polymerization with the addition of H_2_SO_4_ under basic conditions [39]. The addition of PEDOT:PSS improved the coagulation of organic hydrogel solution chains through ionic salt formation. The broad peak at 3450 cm^−1^ for alginate and the alginate/PEDOT:PSS hydrogel is because of the partially overlapping stretching vibrations of the OH groups at high concentrations of PEDOT:PSS (5 mL) and NH groups in the gel state (Figure 3c). The peaks at 2606 and 2400 cm^−1^ are due to the stretching vibrations of saturated C-H bonds, and the peaks at 1623 and 1420 cm^−1^ correspond to the vibration of symmetric and asymmetric stretchings, respectively, of the bonds of -COO− groups in alginate [40]. The peak around 1700 cm^−1^ is due to C=O. The peak at 1060 cm^−1^ is caused by the stretching vibrations of the C-O-C groups, and the peak at 1010 cm^−1^ is due to the -O-C-O- stretching vibrations of alginate. Some peaks disappeared in the FTIR spectrum of the PEDOT: PSS composite with respect to the alginate hydrogel spectrum [41]. The peak at 1620 cm^−1^ for alginate/PEDOT:PSS is attributed to the expansion of vibrations of -C=N groups, which are produced through the chemical reaction of -OH in oxidized starch and H_2_N^-^ in starch from wheat. In addition, the peak at 1326 cm^−1^ is attributed to alginate with bending oscillations -CH,–SO_3_ or -N-CO-CH_2_- groups, which are from alginate/PEDOT:PSS [33,34,35,36,37,38,39,42].

### 2.5. X-ray Diffraction Analysis (XRD)

**Figure 4:**
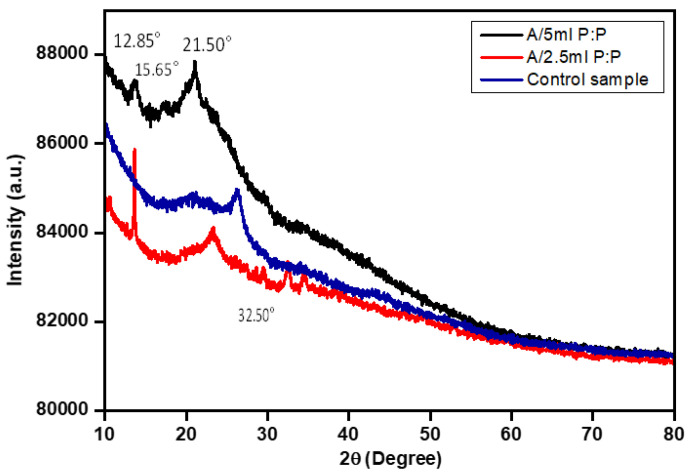
Relative intensity XRD plot of the alginate and A/P:P hydrogel.

The XRD patterns of all porous A/P:P were compared with that of the alginate hydrogel (control) sample, as shown in Figure 4. The presence of PEDOT:PSS in the alginate hydrogel can be clearly observed at A/P:P ratios of 12.85°, 15.65°, 21.50°, and 32.50°); their corresponding Miller indices are (110), (210), (211), (310), and (121), respectively. These three major peaks are known as amorphous corona diffraction of PSS and interlaced packing of PEDOT [43]. All hydrogels are amorphous, but exhibit wide halos in their diffraction patterns, and the difference in the concentration ratio of PEDOT:PSS characterizes components with diffraction features located in different places and at different angles of scattering. The samples treated with PEDOT:PSS at a concentration of 5 mL appear in the form of triangles. Moreover, the peaks decrease in intensity in the hydrogel sample, and the increase in PEDOT:PSS in the pure hydrogel corresponds to the increase in its peak.

The shift of the peaks may be due to the conjugation of the hydrogel with PEDOT:PSS. The samples did not show any large crystalline peaks, owing to the amorphous structures of the alginate hydrogel [44]. The low intensity of PEDOT:PSS (2.5 and 5 mL) indicates a decrease in the degree of crystallization because of the incorporation and interactions between the alginate hydrogel and PEDOT:PSS [45].

### 2.6. Electrochemical Studies of Alginate/PEDOT:PSS Hydrogel Conductive Coaxial and Galvanostatic Charge Discharge (GCD)

To further evaluate the applicability of the hydrogel electrolyte composites for supercapacitors, their specific capacitances, spin performances, and electrochemical stabilities were analyzed. A synthesized hydrogel electrolyte was inserted between the electrodes, and cyclic voltammetry (CV) was performed. The CV curves obtained by the alginate/PEDOT:PSS hydrogel electrolytes (by scanning at various scan rates) are rectangular in shape at low scan rates, but become paper-like with increasing scan rate. The decrease in specific capacitance at higher scanning rates is due to the incomplete reaction of the electrolyte and the rapid movement of ions.

**Figure 5:**
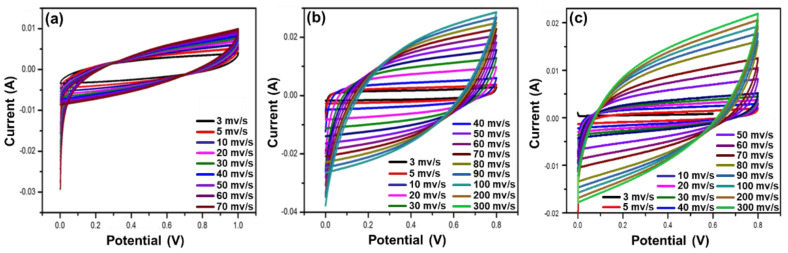
(**a**) CV curves of pure alginate hydrogel; (**b**) CV curves of pure alginate hydrogel with 2.5 mL PEDOT:PSS; and (**c**) CV curves of conductive alginate hydrogel with 5 mL PEDOT:PSS.

Figure 5a shows the CV results of the supercapacitor cells fabricated using the pure alginate hydrogel electrolytes. All cells are observed to maintain their rectangular shape even at higher scanning rates. Among the cells, the pure alginate hydrogel has a rectangular shape at low scanning rates. The maximum specific capacitance of this cell is 3 mV/s, which decreases with increasing scan rates. Figure 5b shows that the maximum specific capacitance for A/P:P with 2.5 mL PEDOT:PSS decreases at a higher scan rate of 300 mV/s. In Figure 5c, supercapacitors made of alginate/PEDOT:PSS show a stable rectangular shape at higher scanning rates and a rate performance of 50% when the scan rate was increased to 300 mV s^−1^. The specific capacitance (*C_s_*) of the conductive A/P:P hydrogel can be calculated according to Equation (1) [46]:

(1) $$C_{s}=\frac{I∆t}{∆V_{m}}$$

where *I* is the charge/discharge current, ∆*t* is the discharge time, ∆*V* is the electrochemical window, and *m* is the mass of the active material within the electrode [47].

The electrochemical behavior of the hydrogel treated with PEDOT:PSS (Figure 5c) shows slight differences in the shapes of the CV curves due to the high mobility of ions and electrons in A/P:P. In Figure 5b, a small change in the shape of the curve and a decrease in the capacitance of the electrolyte treated a ratio of 2.5 mL PEDOT:PSS indicates that the equivalent series resistance is low.

The results showed that the maximum specific capacitances of the alginate hydrogel with 2.5 and 5 mL PEDOT:PSS were 260 and 356 F/g at 100 mV/s, with energy density values of 36.15 and 40.08 W-h/kg at power densities of 100.05 and 400.35 W/kg, respectively. The observed specific capacitance for CV was superior to that reported for PEDOT: PSS-based polymer electrolytes. These improvements are important for practical applications. In Table 1, a comparison of the reported work on hydrogels with the results in the present work is presented.

**Figure 6:**
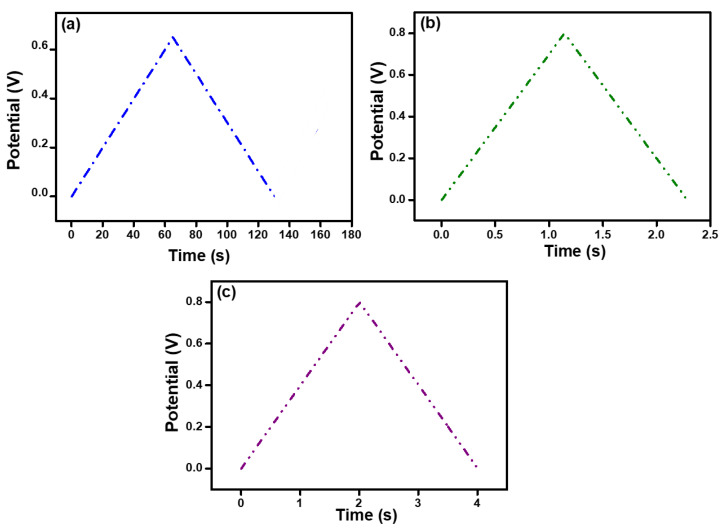
Galvanostatic charge–discharge (GCD) curves at 100 mA/g of (**a**) pure alginate hydrogel, (**b**) alginate hydrogel with 2.5 mL PEDOT:PSS, and (**c**) alginate hydrogel with 5 mL PEDOT:PSS.

The performance of the hydrogels was tested using the GCD technique with a current density range of 30–200 mA g^−1^; the results are shown in Figure 6a,b. The figures show a discharge time that is equal to charge time. This is because of the presence of PEDOT:PSS, which facilitates the transport of charge carriers and increases the surface area. It can be inferred from the GCD curves that the electrode materials are reversed during the charging/discharging process, and the symmetrical curves indicate that the charging and discharging processes of the electrode materials are highly reversible.

**Figure 7:**
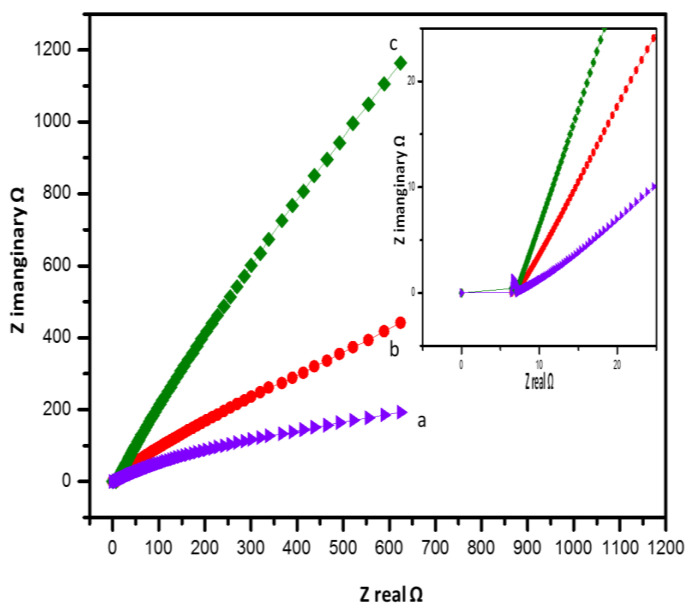
Nyquist plots of the (**a**) pure and (**b**,**c**) A/P:P composite hydrogel electrolytes.

To obtain further information about the capacity of the conductive alginate/PEDOT:PSS hydrogel in supercapacitors, EIS measurements were performed, as shown in Figure 7. The ionic conductivity of the conductive A/P:P hydrogel electrolyte was determined at 25 °C. Among all the synthesized hydrogel electrolytes, PEDOT:PSS made the hydrogels ionically conductive, as it enhanced the ionic conductivity owing to increased ionic diffusion across the hydrogel networks [53,54].

**Figure 8:**
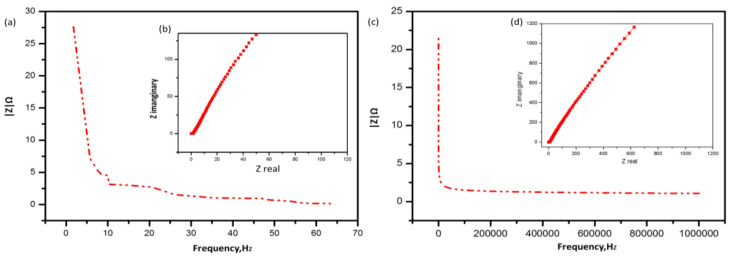
(**a**,**c**) Nyquist plots and (**b**,**d**) Bode plot of the conductive Alginate/PEDOT:PSS hydrogel electrolytes.

It can be seen in Figure 8a–c that the Nyquist plot confirms the capacitive behavior of A/P:P, with lower impedance at high frequencies and a gradient at low frequencies [55,56]. The room-temperature ionic conductivity of the as-prepared alginate hydrogel was found to be 10.5 × 10^−3^ S cm^−1^. In Figure 8b,c, we note a high ionic conductivity, where conductive A/P:P composite hydrogels with 2.5 and 5 mL of PEDOT:PSS achieved maximum ionic conductivities of 11.2 × 10^−3^, and 13.7 × 10^−3^ S cm^−1^, respectively. This is because of the smooth transfer of charge carriers and electrolytic ions through water molecules around the charged groups [57], where the bulk resistance was calculated and the ionic conductivity was determined by means of the following equation:

(2) $$\sigma =L/R_{b}A$$

where σ is the ionic conductivity (S cm^−1^), and L and A refer to the thickness (cm) and active area (cm^2^), respectively.

### 2.7. Electrical Characterization Demonstrating the I-V

**Figure 9:**
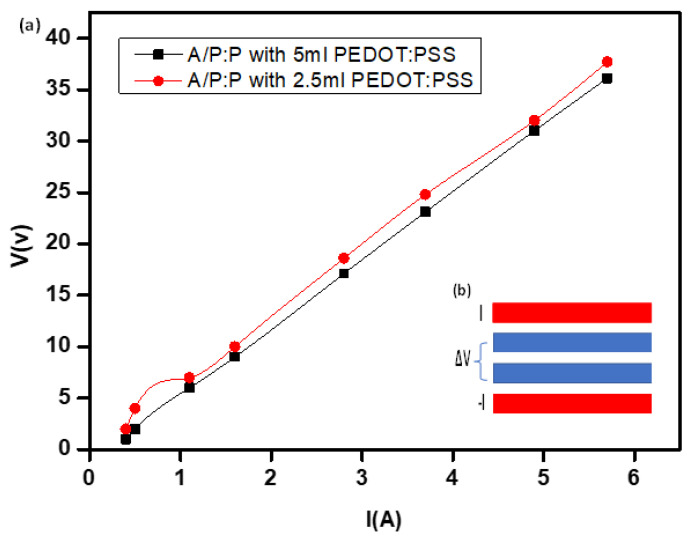
(**a**) I-V curve used for (A/P:P) at concentrations 2.5 and 5 mL of PEDOT:PSS. (**b**) Contact geometry for four-line probe where current I is injected and removed by the two outer contacts, and potential difference ΔV is measured by the two inner contacts.

The hydrogel resistance (R) decreased with an increase in the PEDOT: PSS concentration. Increasing the ratio of PEDOT:PSS improves the electrical characteristics, as shown by the sharpest bend in Figure 9. This is most likely due to the electrophoretic method given by PEDOT-PSS to the pure non-conductive hydrogel [58]. This may be because the higher electron diversity and electrical conductivity along the conductive hydrogel chains provided more efficient electron exchange. The 3D (A/P:P) compound treated with 5 mL PEDOT:PSS exhibited the lowest resistance [38].

**Figure 10:**
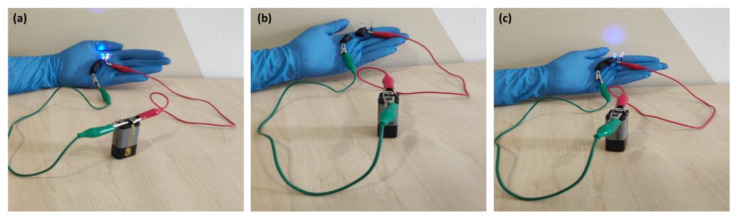
(**a**) LED is illuminated by a conductive hydrogel, (**b**) hydrogel being cut in half, and (**c**) hydrogel reinstated within 10 min of self healing.

The sheet resistance of the conductive hydrogel (A/P:P) was determined, as shown in Table 2. All samples were cross-sectioned with an area of 1 × 1 inches, and estimations were performed at room temperature using the four-line probe technique [59]. The imposed currents created a variable surface potential based on a four-line probe. This is a sufficiently robust and sensitive method for the determination of quartz. Hence, it was used to explore the spatial differences in the electronic conductivity and contact resistance of hydrogels (Figure 9b). Moreover, the four-line probe provides a larger contact area between the probe and A/P:P, while maintaining a small spacing between the lines that serve as the contacts. As shown in Figure 10, the current I and potential difference ΔV are measured, and the apparent resistance R is calculated using Ohm’s law R = ΔV/I [60].

The linear relationship indicates the ideal conductivity in the case of high-concentration PEDOT:PSS, and we note the formation of a straight line passing through the origin. The A/P:P opposition was determined from the condition R_s_ = R (w/d), where w is the width of the sample (w = 2.5 cm) and (d = 0.35 cm) is the line separation. As shown in Table 2, the conductive hydrogel exhibits a pattern similar to that of a stabilizing element. The inclusion of a small amount of PEDOT:PSS (2.5 mL) changes the pure hydrogel to a conductive state because of the arrangement of the associated PEDOT:PSS chains in the hydrogel mixture. The sheet resistance of the A/P:P samples diminishes from 3.5 to 0.9 Ω/square as the concentration of PEDOT:PSS increases from 2.5 to 5 mL of PEDOT: PSS [54].

The gel membrane formation process of organic PEDOT:PSS results in the formation of a 3D fibrous network, which plays a pivotal role in electrical conduction by providing efficient pathways for electrical conduction [55] treated with H_2_SO_4_. It can remove a part of the insulating PSS from the composite hydrogel A/P:P, which significantly improves the conductivity of the composite [61]. These changes in the estimations of sheet opposition can be attributed to the auxiliary dopant (H_2_SO_4_), which causes morphological changes. These changes include expanding PEDOT-rich grains and producing a more slender PSS hindrance by specifically expelling PSS from the PEDOT:PSS film, prompting better charge transport pathways and associations between PEDOT-rich grains [38,58,59,60,61,62]. Moreover, H_2_SO_4_ can also perform PEDOT:PSS installation on the hydrogel sample at room temperature to form a deeply regulated permeation conductive penetration by optimizing the bonds between the PEDOT:PSS chains and the hydrogel [63].

### 2.8. Self Healing and High Electrical Performance

Self healing is a desirable property that is evident in hydrogels possessing ionic bonds. These ionic bonds can be disturbed by stress, which can be reversed by electrostatic interactions between different charged components of ionic bonds, bridging the disruptions and leading to self-healing properties [38,53,54,55,56,57,58,59,60,61,62]. Herein, the self-healing properties of the as-prepared conductive A/P:P hydrogels were evaluated. The conductive A/P:P hydrogel possesses dynamic ionic interactions between the A/P:P carboxyl groups and hydrogen bonds, which are negatively charged gene chains formed by divalent ions [64]. In this study, a piece of conductive A/P:P hydrogel was used as a component of the circuit, and its functionality with respect to conductivity was tested. The results are shown in Figure 10a [65]. However, in step 2 (Figure 10b), realistic evidence of the self-healing ability of the hydrogel was demonstrated, wherein the hydrogel was cut in half with a scalpel. The separated pieces were then placed in contact to allow for independent self healing. It was observed that the pieces fused into one bulk piece again in less than 10 min [66]. Moreover, the ability of the self-healed hydrogel to illuminate the LED was demonstrated (Figure 10c), because the high ionic conductivity of the hydrogel was reinstated upon self healing, and was almost identical to σ = 13.7 × 10^−3^ S cm^−1^. A comparison of the self-healing and high-electrification performances of supercapacitors with other devices described in the literature is shown in detail in Table 3.

**Figure 11:**
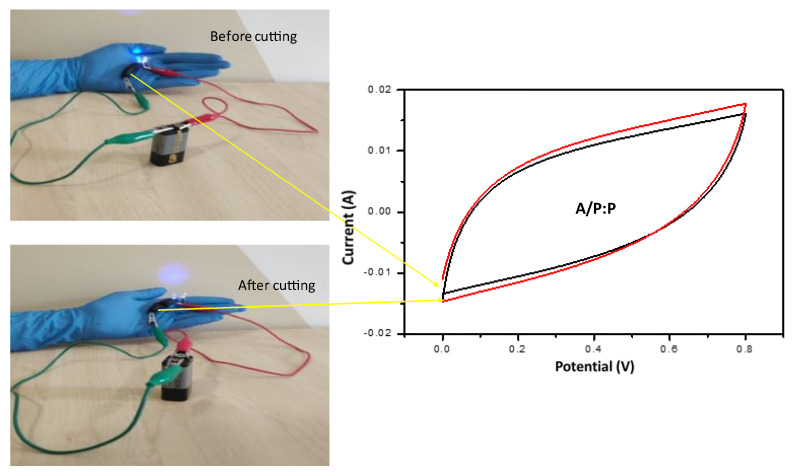
CV curves of self-healed conductive A/P:P hydrogel with high concentration of PEDOT:PSS, the black curve demonstrates the stress before cutting and the red one after cutting.

Figure 11 shows the mechanical strength of the hydrogel before cutting by observing the self-healing efficiency when turning on the LED. When reconnecting the separate ends of a compound A/P:P with the ability to self heal, non-covalent interactions are formed again. LED illumination after the cut pieces were rejoined, providing strong evidence for the self-healing properties of the composite hydrogel. The electrochemical performance was tested by CV before and after cutting the composite A/P:P hydrogel. Furthermore, the CV results demonstrated that the supercapacitor properties of the self-healing conductive A/P:P hydrogels were intact and reinstated once the healing process was completed. 

**Figure 12:**
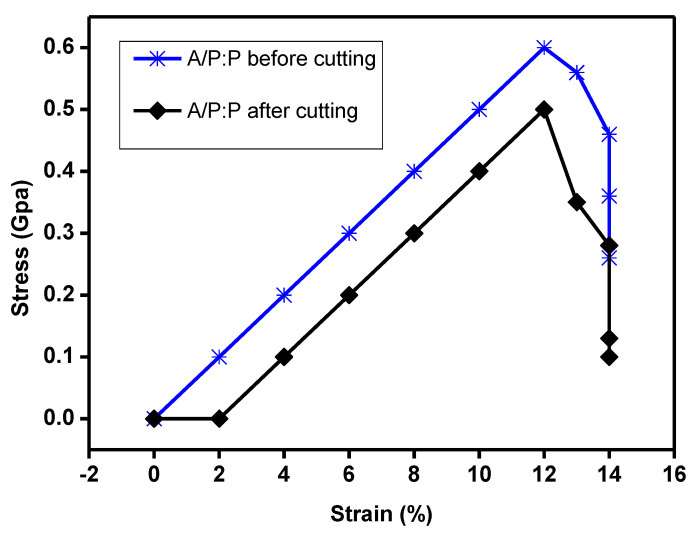
Tensile mechanical properties of hydrogel samples. Typical stress–strain curves for hydrogels and variation in elongation at fracture, hardness, and initial tensile modulus of A/P:P hydrogel with high concentration of PEDOT:PSS before cutting and after cutting, and the maximum stress with different values of x, respectively.

The stress–strain response curves at 300 K along the armchair and zigzag thrusts are shown in Figure 12 relative to the precut specimen. The stress–strain behavior shows a linear correlation that approximately reaches the breaking strength point and terminates with decomposition, resulting in a brittle fracture of the sheet. However, for the sample after self healing, we notice a zigzag curve owing to the cut. Because of the elastic response of the bonds, the stress first shows a linear increasing trend with the stress, and then the bond angle and bond elongation change owing to the combined effect. The stress then exhibits nonlinear elasticity up to the critical value of stress. However, after self healing, we observe a phase shift that led to a simple non-uniform behavior–stress for oriented aliasing [67].

Hydrogel materials such as HPAAN/PDA have shown self healing times of 5 h and mechanical property recoveries of 67% in terms of tensile strain and 78% in terms of modulus [68,69,70].

**Table 3:** Comparing the self-healing and high-electrification performance of supercapacitors with other devices in the literature.

Fabrication	Method	Capacitance/Conductivity	Ref.
**Poly(3,4-ethylenedioxythiophene) (PEDOT) and alginate**	Making multifunctional conductive hydrogels using water-mediated self-assembly and polymerization techniques at room temperature for use as electrodes in supercapacitors. Because both polymers overlap, it is possible to combine the hydrogel’s inherent biocompatibility and sustainability with its flexibility and self-healing abilities. The overlapping of both polymers allows flexibility and self-healing properties to be combined within the same hydrogel with the intrinsic biocompatibility and sustainability of these materials. The intercalation of both polymers allows the flexibility and self-healing properties to be combined within the same hydrogel with the intrinsic biocompatibility and sustainability of these materials. The electrochemical properties do not change significantly after several cut-off/self-healing cycles as observed by cyclic voltammetry. The electrochemical properties do not change significantly after a large number of cut-off/self-healing cycles, as observed by cyclic voltammetry.	Excellent capacitance values (35 mF cm^−a^)	[71]
**Poly (N-acryloyl glycinamide-co-2-acrylamide-2-methylpropanesulfonic) (PNAGA-PAMPS) hydrogels with PEDOT/PSS**	Simply through in situ doping, the specific conductivities of the hydrogels could be significantly enhanced by the doping of PEDOT/PSS. Due to the dynamic breakup and reconstruction of hydrogen bonds, cyclic heating and cooling may result in a reversible sol–gel transition and self-healing ability. The repaired hydrogels successfully regained conductivities as well as mechanical properties.	Conductivity values varies from 0.2 Sm^−a^ to 2.2 Sm^−t^	[72]
**Acrylamide (AAm), lauryl methacrylate (LMA), and 4-(6-(3-(6-(4-methyl-4-oxa-1,4-dihydropyrimidin-2-yl) ureido) hexylcarbamoyloxy) butyl acrylate (UPyHCBA)**	A blade was used to cut the plate into two pieces, which were then joined together at 80 °C to study the self-healing capabilities of PU-PCL substrates. In order to perform the bending without fracturing the damaged area, the broken pieces of PU-PCL were reconnected. The tensile strength of a specimen after healing in comparison to its unhealed counterpart is a measure of healing effectiveness.	52.2 to 33.1 F g^−2^	[73]
**CP’s poly (3,4-ethylenedioxythiophene) (PEDOT)**	Conducting polymeric hydrogels (CPHs) have successfully made a comeback as potential contenders for sensors, energy storage, bioelectronics, medical therapies, and treatments for environmental pollution. Due to their flexibility, CPHs offer a better framework for imagining cutting-edge technologies, including flexible electronic devices like supercapacitors. With a wider range of applications and the added benefit of a flexible synthetic process, PEDOT has emerged as a leading material in the field of bioelectronics.	Electrical conductivity (1–10 S cm^−1^)	[74]
**PVA and PEDOT:PSS**	A semi-interpenetrating polymer network (SIPN) strategy was used to create a set of flexible, stretchable, and conductive composite hydrogel composed of As. With increasing PEDOT:PSS content, the hydrogels’ swelling ratio decreased. PVA networks with semi-interspersed PEDOT:PSS exhibited excellent tensile and compression properties as a result of the chemical crosslinking network and interactions between PVA and PEDOT:PSS. The hydrogels’ electrical conductivity increased with increasing PEDOT:PSS content.	Conductive 4.5 × 10^−3^ S m^−1^	[75]
**A sodium alginate/Na_2_SO_4_**	Combines sodium alginate (SA), borax, and hydrogen bonds between the amino acids in the gelatin chains and the SA to create a double-mesh conductive hydrogel. A phase change material (PCM), Na_2_SO_4_ 10H_2_O, was added to CH, which along with the borax’s nucleation effect improved its ionic conductivity and temperature adaptability. CH exhibits an excellent elongation of 305.7% and a quick self-healing behavior in 60 s.	Capacitance of 185.3 F g^–1^; resistance variation of 2.11 Ω	[76]
**Polymer poly (3,4-ethylenedioxythiophene) polystyrenesulfonate (PEDOT: PSS) iota-carrageenan (CRG), polyvinyl alcohol (PVA)**	Composition made of biopolymers through repeated freezing and thawing. The method, which is based on the creation of a polymer matrix after mixing CRG, PVA, and PEDOT: PSS, and the formation of a porous physical gel as a result of freezing and thawing cycles, is environmentally friendly. The resulting substance can swell in physiological solutions as well as distilled water and is stable in water.	Sheet conductivity was 0.01 [S cm^−1^].	[43]

## 3. Experimental Section

### 3.1. Materials

PEDOT:PSS (poly(3,4-ethylenedioxythiophene):poly(styrene sulfonate)) was bought from Osila, USA. Alginate powder (M_n_ = 357, 475, M_n_/M_w_ = 1.392, M/G = 0.32) was obtained from Sigma-Aldrich (St. Louis, MA, USA). Chemically pure sulfuric acid (H_2_SO_4_) was purchased from Sigma-Aldrich (USA). A graphite conductive substrate was used as the electrode substrate, and it was coated with activated carbon (surface area: 1800–2000 m^2^/g, particle size: 5–20 μm) from Sigma Aldrich. Deionized (DI) water was used as the solvent.

### 3.2. Preparation of Self-Healing Alginate/PEDOT:PSS(A/P:P) Hydrogel Electrolyte

**Figure 13:**
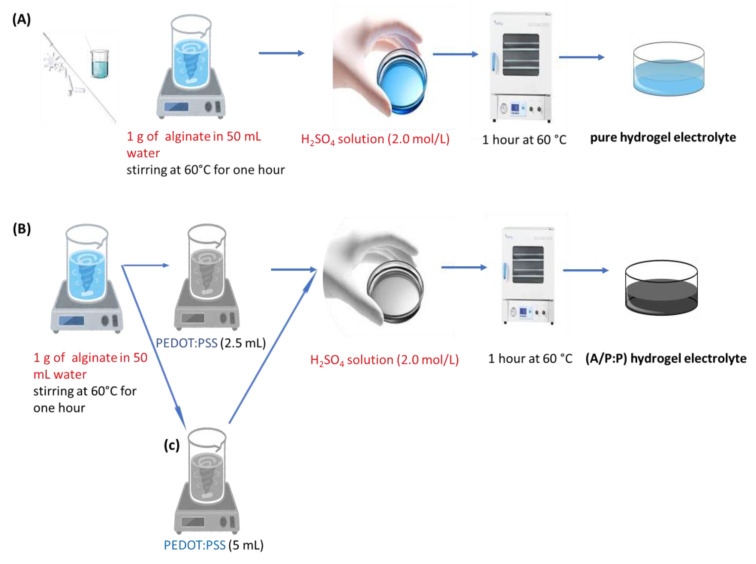
Preparation of (**A**) pure alginate and (**B**,**C**) alginate/PEDOT:PSS (A/P:P) hydrogel electrolytes.

Initially, pure alginate hydrogel was prepared by the following process: 1 g of alginate was dissolved in 50 mL of water, stirred at 60 °C for 1 h using a magnetic stirrer to form a transparent solution. A H_2_SO_4_ solution (2.0 mol/L) was added to start the polymerization reaction. The reaction was carried out for 1 h at 60 °C, as shown in Figure 13A. The self-healable alginate/PEDOT:PSS hydrogel electrolyte was prepared as follows: To begin with, 1 g of alginate was dissolved in 50 mL water, while stirring at 60 °C for 1 h to form a transparent solution. Then, aqueous PEDOT:PSS (2.5 mL) was added to the above solution with vigorous stirring until a bluish-black homogeneous mixture was obtained. The solutions were then added to glass tubes, to which H_2_SO_4_ solution (2.0 mol/L) was added to initiate the polymerization reaction. The reaction was carried out for 1 h at 60 °C, and a hydrogel was obtained (Figure 13B). In Figure 13C, we repeat the same steps as in B, but increasing the PEDOT:PSS ratio to 5 mL. By controlling the appropriate amount of PEDOT:PSS, the order, shape, and coherence of the sample can be maintained. Moreover, the strong bonding between the conductive domains rich in PEDOT:PSS and alginate provides excellent electrical conductivity and aqueous stability. Table 4 lists the detailed compositions of the prepared samples.

PEDOT polymerizes in the presence of alginate to produce porous alginate conductive PEDOT scaffolds, resulting in the synthesis of electrically conductive alginate/PEDOT:PSS within a block matrix using H_2_SO_4_. H_2_SO_4_ causes chemical crosslinking of PEDOT:PSS with alginate. These compounds demonstrate great potential in the fields of tissue engineering and elastomeric reversible materials. This potential arises from their adequate cellular compatibility and simplicity of manipulation through the chemical crosslinking of PEDOT:PSS with alginate as a result of H_2_SO_4_ usage. An alginate/PEDOT:PSS hydrogel was formed when the hydrogel electrolyte was treated with H_2_SO_4_ acid to create 3D hydrogels. This process is called “crystallization”, because it causes the material to acquire a stacked, interlocking nanostructure. In contrast, PEDOT:PSS with sulfuric acid dissolves PSS and produces nanofibers of PEDOT through a structural transition mechanism that resembles grains. The conductivity of the crosslinked alginate/PEDOT:PSS hydrogel can be improved by H_2_SO_4_ crystallization while maintaining its stability and cytocompatibility. PEDOT:PSS is perfect for the crosslinking solidification process, which allows for the control of pore size, quantity, and orientation because it is water-dispersible. Additionally, the hydrogel exhibited significantly improved flexibility and conductivity, while maintaining biocompatibility. By extending the binary system, PEDOT: PSS-based ternary composite films can become high-performance materials with several benefits. When the nanoparticles were dispersed in PEDOT:PSS solutions, they formed dense interlocking channels that decreased the interchain distance and consequently increased the conductivity of the resulting film. PEDOT:PSS primarily serves as a binder to encapsulate binary hybrids. High-strength supramolecular polymer conductive hydrogels were created that were crosslinked by a single amide hydrogen bond and strengthened [6,77,78].

### 3.3. Fabrication of Electrode

Carbon slurry was used to construct the working electrode. This slurry was prepared by adding 80:10:10 mass percentages of activated carbon, carbon black, and poly (vinylidene fluoride) binder, respectively, to N-methyl-2-pyrrolidone solvent. To ensure homogeneity, the mixture was stirred continuously for a few hours at RT. The slurry was then applied to the activated carbon and left to dry for 1 h at 100 °C.

### 3.4. Fabrication of Smart Flexible Electrochemical Supercapacitor

The working electrode substrate was prepared as follows:

A conductive graphite substrate was coated with activated carbon black by dispersing an appropriate amount of graphite sheets. The electrode substrate was obtained by drying for 1 h at a temperature of 100 °C. For the assembly of the bipolar supercapacitor device, the hydrogel was first squarely cut to a size of 2 cm × 2 cm and used simultaneously as an electrolyte and separator. Subsequently, a smart flexible supercapacitor was assembled by sandwiching the hydrogel electrolyte between the prepared electrodes (electrode/hydrogel electrolyte/electrode).

### 3.5. Characterization

#### 3.5.1. Structural and Morphological Analyses

The surface morphology was studied using field-emission scanning electron microscopy (SEM (JEOL, JSM-6380LA)) with an accelerating voltage of 5 kV. The samples were coated with gold before conducting SEM analysis. The structural characterization of the pure and synthesized hydrogels was performed using Fourier-transform infrared spectroscopy (FTIR). A Nicolet iS50 FTIR spectrometer (Thermo Fisher Scientific Co. (Waltham, MA, USA)) was used to record the FTIR spectra of the pure and synthesized alginate hydrogels. The spectral range and resolution factor of the FTIR spectrometer ranged from 500 to 4000 cm^−1^. The X-ray diffraction (XRD) patterns of the porous alginate hydrogels and A/P:P were obtained using Cu-Kα radiation (1.5406 Å) at a current of 30 mA, voltage of 40 kV, and scanning angle 2θ over a step size of 0.02/s.

#### 3.5.2. Electrochemical Studies of Electrolyte and Supercapacitor

The ionic conductivity (σ) of the alginate/PEDOT:PSS hydrogel electrolyte was estimated using an impedance spectroscopic instrument Hioki 3532-50 LCR HiTESTER over a frequency range of 50 Hz to 5 MHz. Electrochemical studies were performed for electrochemical cells, which were prepared by sandwiching the as-prepared polymer hydrogel (alginate/PEDOT:PSS) electrolyte between activated carbon materials coated with graphite electrodes. CV, galvanostatic charge–discharge (GCD), and electrochemical impedance spectroscopy (EIS) on alginate/PEDOT:PSS hydrogel electrolytes were performed using a Gamry Interface 1000. To investigate the electrical properties of the conductive hydrogels, a four-line probe method was used. The electrical resistance of the hydrogel synthesized from I-V bends was determined at a temperature of 25 °C. The electrical resistance (R) of each gel was measured during drying using a four-line probe method. The resistance of the plate was determined using the following equation [42]:

(3) $$R_{s}=R\left(\frac{w}{d}\right)$$

where w is the width of the sample (2.5 cm) and d is the spacer between the leads (0.35 cm).

#### 3.5.3. Tensile Test

Tensile testing was performed using an Instron 3360 electronic universal testing device (Instron Corporation, Norfolk County, MA, USA). The prepared specimens were cut into rectangular shapes according to the JISK6251-7 standard size (length 2 cm, width 0.5 cm, gauge length 0.5 mm).

## 4. Conclusions

In summary, the present study aimed to analyze the self-healing properties of semi-IPN hydrogels based on PEDOT:PSS with the additive H₂SO₃. We developed a facile and scalable protocol for the preparation of conductive flexible alginate/PEDOT:PSS hydrogels with tunable 3D microstructures as electrically active materials for high-performance flexible solid-state supercapacitors. A/P:P hydrogel capacitors were successfully synthesized by employing H_2_SO_4_ as an initiator to yield a polymeric alginate hydrogel with PEDOT:PSS encapsulated within the matrices, which was employed as an electrolyte for the supercapacitor. Analysis of the structure and surface morphology of the pure and synthesized hydrogels were performed using Fourier-transform infrared (FTIR) spectroscopy and scanning electron microscopy (SEM), respectively. The symmetric alginate with 5 mL of the PEDOT: PSS-based flexible supercapacitor achieved a specific capacitance of 356 F/g at 100 mV/s (energy density of 40.08 Wh/kg at a power density of 400.35 W/kg) at 100 mV/s g^−1^ and sheet resistance of 0.9 Ω/square. The ionic conductivity was measured at 25 °C and was found to be high, i.e., 13.7 × 10^−3^ S cm^−1^. The as-prepared alginate/PEDOT:PSS hydrogel displayed conductive properties, and had the potential to achieve rapid self healing.

## Figures and Tables

**Table 1:** Comparison of the electrochemical performance of the assembled supercapacitor with the previous work.

S. No	Materials	Applications	Capacity	Ref.
**1.**	Alginate/PEDOT:PSS	An energy storage device in portable and wearable electronic devices	Areal capacity 567.6 F.cm^−3^ and 1002.4 mF.cm^−2,^ large specific capacitance 246.4 mF.cm^−2^, high energy density of 21 μW.h.cm^−2^	[48]
**2.**	Soft alginic acid gel/PEDOT/PSS coated multi-walled carbon nanotubes (MWCNT)	Supercapacitors.to modify the neural interface	Capacity of charge transfer observably increased from 0.5 mC/cm^2^ to 1.2 mC/cm^2^	[49]
**3.**	PEDOT polymerized on N-doped carbon nanofibers (CNFs)	Supercapacitors	Exhibited a capacitance of 203 F g^−1^, nearly reaching its theoretical capacity of 210 F g^−1^, energy density of 97 μW-h cm^−2^	[50]
**4.**	PEDOT: PSS hydrogel film	Supercapacitors	158 F g⁻¹ at a scan rate of 50 mV s⁻¹, cycling stability with 84.9% and capacitance retention after 2000 cycles.	[51]
**5.**	Alginic acid sodium salt/PEDOT:PSS	An aqueous supercapacitor	Exhibited high conductivity of 168.4 mS cm^−1^	[52]
**6.**	Alginate/PEDOT:PSS	A stable gel electrolyte membrane	356 F g^−1^ at 100 mv/s g^−1^.	This work

**Table 2:** Sheet resistance of (A/P:P) at different concentrations of PEDOT:PSS.

Rs (Ω/Square)	Alginate Coating
**3.5**	2.5 mL PEDOT:PSS
**0.9**	5 mL PEDOT:PSS

**Table 4:** Details of the A/P:P with respect to the PEDOT:PSS ratio.

System	Details
A-Pure alginate hydrogel	(1 g alginate + 50 mL of water) + 2.0 mol/L H_2_SO_4_
B-A/P:P	2.5 mL PEDOT:PSS + pure alginate hydrogel solution+2.0 mol/L H_2_SO_4_.
C-A/P:P	5ml PEDOT:PSS + pure alginate hydrogel solution+2.0 mol/L H_2_SO_4_.

## Data Availability

Data is contained within the article.
